# An Efficient Procedure for Bonding Piezoelectric Transducers to Thermoplastic Composite Structures for SHM Application and Its Durability in Aeronautical Environmental Conditions

**DOI:** 10.3390/s23104784

**Published:** 2023-05-16

**Authors:** Tasdeeq Sofi, Maria R. Gude, Peter Wierach, Isabel Martin, Eduardo Lorenzo

**Affiliations:** 1 Foundation for the Research, Development and Application of Composite Materials (FIDAMC), Avda. Rita Levi Montalcini 29, Tecnogetafe, 28906 Getafe, Spain; maria.r.rodriguez@fidamc.es (M.R.G.); maria.i.martin.external@fidamc.es (I.M.); eduardo.lorenzo@fidamc.es (E.L.); 2 Institut für Polymerwerkstoffe und Kunststofftechnik TU Clausthal, Agricolastraße 6, 38678 Clausthal-Zellerfeld, Germany; peter.wierach@dlr.de; 3 German Aerospace Center (DLR), Lilienthalplatz 7, 38108 Braunschweig, Germany

**Keywords:** composite materials, acousto-ultrasonic transducers, thermoplastic adhesive films, sensor bonding, electro-mechanical susceptance, durability and integrity, structural health monitoring (SHM)

## Abstract

Piezoceramic transducers (PCTs) bonded to carbon fiber-reinforced plastic (CFRP) composite structures must be durable as well as remain properly bonded to the structure in order to provide reliable data for accurate guided-wave-based structural health monitoring (SHM) of aeronautical components. The current method of bonding transducers to composite structures through epoxy adhesives faces some shortcomings, such as difficult reparability, lack of weldability, longer curing cycles, and shorter shelf life. To overcome these shortcomings, a new efficient procedure for bonding the transducers to thermoplastic (TP) composite structures was developed by utilizing TP adhesive films. Application-suitable TP films (TPFs) were identified and characterized through standard differential scanning calorimetry (DSC) and single lap shear (SLS) tests to study their melting behavior and bonding strength, respectively. Special PCTs called acousto-ultrasonic composite transducers (AUCTs) were bonded to high-performance TP composites (carbon fiber Poly-Ether-Ether-Ketone) coupons with a reference adhesive (Loctite EA 9695) and the selected TPFs. The integrity and durability of the bonded AUCTs in aeronautical operational environmental conditions (AOEC) were assessed in accordance to the standard Radio Technical Commission for Aeronautics DO-160. The AOEC tests performed were operating low and high temperatures, thermal cycling, hot-wet, and fluid susceptibility tests. The health and bonding quality of the AUCTs were evaluated by the electro-mechanical impedance (EMI) spectroscopy method and ultrasonic inspections. The AUCT defects were created artificially and their influence on the susceptance spectra (SS) was measured to compare them with the AOEC-tested AUCTs. The results show that a small change occurred in the SS characteristics of the bonded AUCTs in all of the adhesive cases after the AOEC tests. After comparing the changes in SS characteristics of simulated defects with that of the AOEC-tested AUCTs, the change is relatively smaller and therefore it can be concluded that no serious degradation of the AUCT or the adhesive layer has occurred. It was observed that the most critical tests among the AOEC tests are the fluid susceptibility tests, which can cause the biggest change in the SS characteristics. Comparing the performance of the AUCTs bonded with the reference adhesive and the selected TPFs in the AOEC tests, it was seen that some of the TPFs, e.g., Pontacol 22.100 outperforms the reference adhesive, while the other TPFs have similar performance to that of the reference adhesive. Therefore, in conclusion, the AUCTs bonded with the selected TPFs can withstand the operational and environmental conditions of an aircraft structure, and hence, the proposed procedure is easily installed, reparable, and a more reliable method of bonding sensors to aircraft structures.

## 1. Introduction

Carbon fiber-reinforced plastic (CFRP) composite materials are high-performance materials that are increasingly used as a primary material for engineering structures in the aerospace, wind energy, naval, and other industries. Due to their extraordinarily high mechanical performance, reduced weight, and high durability, as compared to traditional materials such as aluminum or steel, there is growing use of these materials in structural applications [1]. These advantages combined with the current emerging challenges, such as climate change and ensuring the sustainability of the technological development, make the employment of these types of composite materials more desirable within modern industries.

Composite structures are vulnerable to impact damage during operation, especially barely visible impact damages. They may exhibit a variety of structural failure cases, such as delamination, fiber breakage, and matrix cracking, among others [2]. The mechanics of this complex and multivariate damage is difficult to predict, which can lead to uncertainty in the assessment of current and future material behavior. Therefore, detailed periodic inspection needs to be carried out on these structures to avoid any possible structural failure. These schedule-driven inspections and maintenance periods leads to significant costs as well as the nonavailability of the assets during these periods [3]. Furthermore, the structural integrity between the two maintenance schedules is unknown, and therefore, it can sometimes lead to unnecessary and other times late maintenance.

Structural Health Monitoring (SHM) offers a paradigm shift from a schedule-driven maintenance to a condition-based maintenance approach. It provides a rational approach by enabling cost-effective risk-based maintenance schedules based on predictions of the remaining useful life as opposed to periodic inspections. SHM is defined as a process of collecting and analyzing data from a distributed network of sensors that are mounted on or embedded inside a structure to obtain information about the health of the structure [3]. In recent years, SHM has emerged as an interesting technology for many industries because of its capability to reduce maintenance costs, and therefore, reduce labor and downtime with an increment of the operational availability. However, despite these advantages, SHM systems are still not reflected in the real-life applications as expected because of the lack of industry practices, guidelines, and standards that the system must fulfill in different conditions, particularly in the aerospace industry. Among the different SHM approaches, guided.

SHM using piezoceramic transducers (PCTs) has relatively higher maturity and is being widely used for SHM of composite structures [4]. It is one of the most active fields of research for aeronautic structures because it has a very high resolution as demanded by the aeronautical industry [5]. An extensive literature review on guided-wave-based SHM can be found in [5,6,7].

One of the challenges in the upscaling of guided-wave-based SHM in aerospace structures is the fast and cost-efficient integration of PCTs to composite structures. Generally, two methods are used to integrate the PCTs with the structures, namely co-bonding and secondary bonding [8,9,10]. In co-bonding, transducers are integrated during the manufacturing of the host structure itself, while in secondary bonding, the transducers are integrated with the host structure in a separate step after its manufacturing. In co-bonding, the transducers can be embedded within the structure or surface-mounted, while in secondary bonding, they are always surface-mounted. Co-bonding is not desired in case of high-performance thermoplastic (TP), such as Poly-Ether-Ether-Ketone (PEEK) or Poly-Ether-Ketone-Ketone (PEKK), because the PCTs will get depolarized in the high-temperature manufacturing conditions for these types of composite materials. The main focus of this work is the integration of PCTs to high-performance TP-based composites in general but carbon-fiber PEEK (CF-PEEK) composites in particular. The use of these TP composite materials has been growing in the aeronautical industry because of some of the advantages they have as compared to thermoset-based composites, such as easier recyclability, weldability, infinite shelf life, and ability to be stored at room conditions. In addition, they have better performance in aeronautical operational environmental conditions (AOEC) due to properties such as lower moisture absorption [11,12]. The state-of-the-art for bonding PCTs to TP composite structures is through secondary bonding by using high-performance epoxy adhesives [9]. However, many disadvantages are faced for epoxy adhesive, such as difficult repair procedure, long curing cycles, and storage and essential refrigeration conditions. Therefore, there is a need to develop a new approach for integrating sensors to TP-based composites. These disadvantages faced by thermoset adhesives can be overcome by using TP-based adhesive films, as shown in Table 1. In addition, TP-based adhesives are better suited to TP-based composites. Furthermore, new efficient and fast methods of bonding PCTs or sensors in general to composite structures can be developed, such as using electromagnetic induction heating or simply using heat to melt the TP adhesive, while the PCT and the structure are in contact under pressure, e.g., roller pressure. Therefore, the proposed use of TP films (TPFs) is one of the strategies towards this new approach and it would allow easier and efficient integration of sensors to TP composite structures.

During operation of an aircraft, both the PCT material as well as the adhesive layer of the bonded PCTs are prone to damage and degradation [13]. As pointed out in [14], degradation of a PCT is a damage caused by cycling mechanical loading and environmental exposure. Extremely low and high temperature exposure can cause degradation to the adhesive, the PCT material and the encapsulation material of the PCTs [15,16]. Moreover, some adhesives can also get degraded in certain fluids, such as the adhesive Z70 (HBM GmbH, Darmstadt, Germany), can get degraded in dimethylformamide, as reported in [15]. Both high mechanical loading and harsh environmental conditions are encountered in aeronautical applications; therefore, it becomes necessary to evaluate the integrity and durability of the bonded PCTs in mechanical tests and AOEC to assess their durability during the lifetime of an aeronautical structure.

A few studies [8,10,17,18,19] have been carried out to study the integrity and durability of PCT systems integrated to composite structures through co-bonding. In this study, only secondary bonding has been used; therefore, detailed literature review on secondary bonding has been presented only. Adhesives such as fast-acting Cyanoacrylate adhesives [20,21], Hexcel Redux 312 film [22] and Hysol EA9394 and EA9294 [23,24] are some of the adhesives that were used to bond PCTs to composite as well as metallic structures through secondary bonding for SHM. Some of these studies have investigated the integrity and durability of bonded PCTs in different environmental exposures and mechanical tests. Eckstein et al. [23] evaluated the durability of secondary bonded sensors on a real aircraft under realistic aircraft conditions, namely pressurization, vibration, high and low temperature, temperature cycling, and lightning strike. Furthermore, the influence of environmental and operational condition on guided wave propagation was assessed. Marzani et al. [24] investigated the reliability and bonding strength of PCTs as well as defect detection and localization procedures on a composite outer wing demonstrator in fatigue and impact tests. Thielicke et al. [25] studied the reliability and the adhesion of sensor patches bonded to composite and steel substrates with a standard unnamed epoxy under quasi-static bending and cyclic loading. An insignificant amount of damage was reported on the tested sensor patches after the tests. Gong et al. [20] used Cyanoacrylate adhesive and found out that the adhesive is subject to degradation due to moisture absorption in high-temperature and high-humidity conditions. Salmanpour et al. [26] investigated the integrity and durability of DuraAct^TM^ [27] manufactured by PI Ceramic, Lederhose, Germany, “SMART Layer” (Stanford multi-actuator-receiver transduction layers) manufactured by Acellent technologies Inc., Sunnyvale, CA, United States [28], as well as their in-house developed “SHM layer” in airborne conditions. According to the study “SMART layer” failed in across all the test parameters and the authors suspected that the degradation occurred because of the unspecified epoxy resin used by the manufacturer for bonding the PCT discs to the Kapton layer. These studies show that the adhesive plays a significant role in the integrity, durability and reliability of PCT-based SHM systems.

Regarding other type of sensors, such as bonding fiber optic sensors to CFRP structures for aerospace applications, in [29], typical adhesives used for bonding optical fiber to composite structures were compared. Adhesive HBM X120 was selected because of its lower viscosity and flight requirement temperature range. A detailed study on the integrity and durability of fiber optic sensors bonded to composite coupons was evaluated against in-flight conditions. Similarly, in [30] fiber Bragg grating sensors were bonded to composite structures with an unnamed epoxy adhesives and their integrity was tested in different aeronautical conditions.

All of the studies mentioned above have used conventional epoxy adhesives for bonding the PCTs to the host structures. Yue et al. [8,9] is the only study known to the author in which the PCTs were bonded to thermoset as well as TP-based composites using TPFs and their durability was investigated. However, more comprehensive work is required, particularly identification of better performance TPFs as well as evaluating their integrity and durability in AOEC and in different mechanical tests. Therefore, in this study, new TPFs have been identified, characterized by thermal analysis, tested for reparability, and their bonding capability demonstrated and measured. The integrity and durability of the bonded PCTs with the selected TPFs have been investigated in AOEC in accordance to the standard Radio Technical Commission for Aeronautics (RTCA) DO-160. The objectives of this work, therefore, are as follows:

- Obtaining controllable and reliably repeatable bonding quality by using TPFs rather than liquid two-part epoxy adhesives;
- Using TP adhesive films in place of epoxy adhesives, which are irreparable, are often messy, and have long curing cycles;
- Easy replacement of faulty PCTs without damaging or disturbing the host structure in any form;
- Demonstrating the integrity, durability, and reliability of this new bonding procedure in AOEC in accordance to the aeronautical standard DO-160 and comparing the results with a reference epoxy adhesive.

## 2. Experimental Methods

### 2.1. Materials

The TPFs were shortlisted on the following criteria:

- Melting temperature ($T_{m}$) ≤ 150 °C: The $T_{m}$ of the TPFs should be less than the maximum allowable temperature of the PCTs under consideration and should not exceed the glass transition temperature of PEEK.
- Deflection temperature ≥ 70 °C: The deflection temperature of the TPFs should be greater than the service temperature of the host structure (Aircraft structure) or the higher operating temperature as defined by the DO-160 standard for internal electronic components, i.e., 70 °C.
- Good bonding strength: The adhesive bond line should be able to resist the strains and should not fail during the normal loading conditions. Semi-crystalline TPFs will be preferred because of their better mechanical performance and chemical resistance and predictable behavior as compared to the amorphous polymer films [31].

Based on the above-mentioned criteria, in total, thirteen TPFs were identified initially based on the data provided by the suppliers and are listed in Table 2. In order to compare the performance of the selected TPFs, an epoxy film adhesive, namely Loctite EA-9695, was taken as the reference adhesive because it is a high-performance adhesive used in aeronautical bonding applications and has good environmental resistance. The PCTs, namely DuraAct^TM^ [27], were supplied from PI Ceramic. These are special PCTs embedded in a ductile polymer (RTM-6) and provided with a mechanical pre-compression to form an acousto-ultrasonic composite transducers (AUCT) [32]. This pre-compression protects the brittle piezoceramic material and allows tensile loads on the AUCT. The AUCTs were bonded to CF-PEEK (APC-2) as a host material.

Due to electro-mechanical coupling, the electrical properties of the PCT also becomes dependent on the host structure to which it is bonded. The properties of a bonded PCT are, therefore, generally referred as “electro-mechanical” properties. The admittance of a free PCT ($Y_{free}\left(\omega \right)$) is given in Equation (1) as [33]:

(1) $$\begin{array}{c} Y_{free}\left(\omega \right)=G\left(\omega \right)+iB\left(\omega \right)=i\omega \frac{bl}{t_{C}}\left(\epsilon_{33}^{T}\left(1-i\delta \right)\right) \end{array}$$

The admittance of a bonded PCT known as electro-mechanical admittance (EMA) is given in Equation (2) as:

(2) $$\begin{array}{c} Y\left(\omega \right)=i\omega \frac{bl}{t_{c}}\left[\epsilon_{33}^{T}\left(1-i\delta \right)d_{31}^{2}Y_{P}^{E}+\frac{Z_{PCT}\left(\omega \right)}{\left(Z_{PCT}\left(\omega \right)+Z_{s}\left(\omega \right)\right)}d_{31}^{2}\hat{Y_{P}^{E}}\left(\frac{\tan(\kappa l)}{\kappa l}\right)\right] \end{array}$$

where G(ω) and iB(ω) are the real and imaginary part of the admittance and are known as conductance and susceptance, respectively; ω is the excitation angular frequency; $Z_{PCT}\left(\omega \right)$ and $Z_{s}\left(\omega \right)$ are the PCT and the structure impedance, respectively; *b*, *l*, and *t* are the width, length, and thickness of the PCT, respectively; δ and $\epsilon_{33}^{T}$ are the dielectric loss tangent and the dielectric permittivity of the PCT, respectively; $\hat{Y_{P}^{E}}$ is the complex Young’s modulus of the PCT at zero electric field; $d_{31}$ is the coupling constant; and κ is defined as $\omega \sqrt{\frac{\rho }{\hat{Y_{P}^{E}}}}$.

Both electro-mechanical impedance (EMI) or EMA spectra can evaluate the health as well as bonding condition of a PCT and has been used by many studies as an criterion to evaluate the sensor health and debonding [10,15,34,35]. However, the imaginary parts of EMI or EMA are predominantly affected by the PCT defects, and especially the imaginary part of the admittance, known as susceptance, is sensitive to the changes in the bond layer [15,36]. The main characteristic of the susceptance spectra (SS) that were used to evaluate the integrity and durability of the AUCTs during the AOEC tests are as follows:

- The slope of the SS curve obtaining through linear fitting;
- The change in the resonance characteristics of the SS, i.e., the amplitude and the frequency of the first natural frequency. The first resonant peak is used, because the guided waves are generated using this frequency range for the AUCTs under consideration.

The changes in the above performance indices are calculated from the percentage root mean square deviations (RMSDs) calculated after each AOEC test and is defined in Equation (3) as:

(3) $$\begin{array}{c} RMSD\left(\%\right)=\sqrt{\frac{\sum_{i=1}^{n}{\left(G\left(\omega_{i}\right)-G_{0}\left(\omega_{i}\right)\right)}^{2}}{\sum_{i=1}^{n}{\left(G_{0}\left(\omega_{i}\right)\right)}^{2}}}\times 100 \end{array}$$

where ω is the angular frequency, *n* is the number of points, and $G_{0}\left(\omega_{i}\right)$ and $G(\omega_{i})$ are the baseline and the current signal, respectively.

To understand how different AUCT faults affect the susceptance spectra (SS), the defects that can occur on a AUCT during its lifetime were created artificially and their influence on the SS was measured. The measurements were used as a reference in order to compare the post-test SS of the AUCTs under test with that of the faulty AUCTs in order to determine if any defect or degradation had occurred on the AUCTs or the adhesive layer during the AOEC tests. The AUCTs were bonded to CF-PEEK composite coupons with the reference adhesive and three kinds of commonly occurring AUCT defects were created, namely wear-off, breakage, and debonding. The wear-off was created in three grades. In the first grade, some part of the encapsulation of the AUCT was removed such that the piezoceramic was not visible, in the second grade, the encapsulation layer was completely removed such that the piezoceramic became visible, and in the third grade, severe wear-off was carried out by removing the piezoceramic material as well. The debondings were also introduced in three levels, namely 25%, 50%, and 75% of the AUCT was debonded. In case of the AUCT breakage case, only 15% of the piezoceramic was broken and rest was kept healthy and well bonded.

Ultrasonic (US) inspections were performed to further inspect the AOEC-tested AUCTs bonded with different adhesives under consideration. The inspections were carried out with an immersion tank Triton 8000TT+ from TecniTestNDT. The Pulse-echo method was used with a scanning speed of 50 mm/s and scanning index of 1 mm to generate the C-scans registers as well as A-scan for each point of register. The AUCTs were sealed with blue tape so that no water went inside the adhesive layer. Reference scans were taken for the AUCT’s with artificially introduced debonding of 25%, 50%, and 75%, so that they can be compared with the US scans of the AOEC-tested AUCTs and determine if any significant debonding has occurred in the adhesive layer.

### 2.2. Thermal Analysis of Thermoplastic Films

Differential scanning calorimetry (DSC) tests were carried out to study the melting behavior and crystallinity characteristics of all thirteen identified TPFs. Although the melting point ($T_{m}$) was provided by the suppliers, it was necessary to study the melting behavior and crystallinity characteristics through a standard test. The DSC tests were carried on a DSC Q200 TA instruments equipment according to the standard ISO 11357-3. At least three samples were tested for each film.

### 2.3. Transducer Bonding and Reparability

The AUCTs were bonded to CF-PEEK composite coupons through secondary bonding. Only those TPFs were used which had the desired melting behavior as obtained from the DSC tests. Before bonding the AUCTs, the composite surface at the sensor locations was prepared by sanding to increase the surface roughness for better mechanical adhesion and subsequently cleaned and degreased with an alcohol solution. Strips of TPFs were placed at the prepared locations, then the transducers were placed over the film strips and secured through Kapton tape. The whole setup was then put inside a vacuum bag and heated in an oven at a temperature greater than 10 °C of the $T_{m}$ of the corresponding films for 25 min. The setup was allowed to cool under vacuum until room temperature was reached.

The bonding of the AUCTs with the TPFs were evaluated by comparing their post-bonding EMI spectra with that of a free AUCT and sending lamb waves from a AUCT bonded with the reference adhesive to AUCTs bonded with the reference adhesive and two different TPFs. The EMI measurements were taken with C60 impedance analyzer from Cypher instruments over a range of 1 kHz to 1 MHz. The guided waves were transmitted and received using a National instrument PXIe-1082. An actuation signal of the three-pulse sine wave with a Hanning window and amplitude of 3 Volts was applied. The frequency was varied from 40 kHz to 350 kHz in steps of 10 kHz and the signal from the AUCTs was recorded for a duration of 600 µs.

The reparability of the TPFs was demonstrated by heating the adhesive layer of the bonded AUCTs by a temperature-controlled heat gun to the $T_{m}$ and then removing the transducers through an easy procedure without causing any damage or degradation to the host structure whatsoever.

### 2.4. Adhesive Bonding Quality

The single shear lap (SLS) tests were carried out to evaluate the bonding quality of the TPFs on PEEK surface and compare them with the reference adhesive. Only those TPFs were tested that had the desired melting behavior as obtained from the DSC tests and that had passed the reparability tests. In total, eight films were tested, including the reference adhesive. The tests were carried out on a MTS universal testing machine according to the standard EN 2243-1 by bonding CF-PEEK (HTA-40) composite specimen to each other with the selected films. At least three specimen were prepared for each case.

### 2.5. Aircraft Operational Environmental Condition Tests

**Figure 1:**
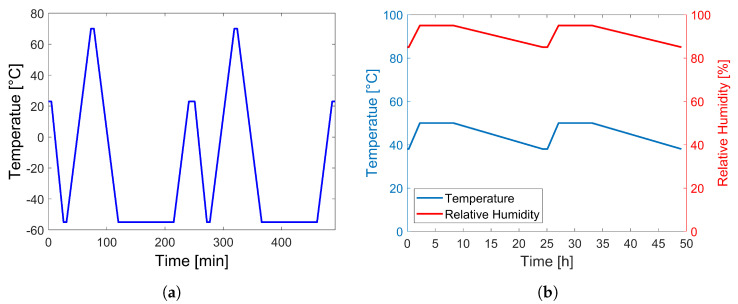
Aeronautical operational environmental conditions (AOEC) tests profiles for (**a**) thermal cycling and (**b**) hot–wet.

The AOEC tests were carried out according to the standard DO-160. The following tests were carried out: low and high temperature, thermal cycling (Figure 1a), hot-wet (Figure 1b), and fluid susceptibility, as described in Table 3. In case of fluid susceptibility tests, the bonded AUCTs were immersed in the test fluids for a minimum of 24 h as per the standard; however, they were not dried in a chamber for 160 h as recommended by the standard, rather they were dried at room temperature for a minimum of three weeks. The AOEC tests were carried out on the AUCTs bonded with the reference adhesive and five TPFs only. The five TPFs were chosen from the initially short listed thirteen TPFs based on the results obtained from the DSC, reparability, and SLS tests. In addition, three free (not bonded) AUCTs were also put through the AOEC tests in order to distinguish between the AUCT and the adhesive layer degradation during the AOEC tests. The AUCTs were bonded to CF-PEEK (APC-2) composite coupons having dimensions of 260×20×1.82 mm${}^{3}$ and a layup sequence of [±45/0${}_{3}$/90/0/90/0${}_{3}$/±45] and three samples were prepared for each case.

## 3. Results and Discussion

### 3.1. Adhesive Characterization

#### 3.1.1. Thermal Analysis

**Figure 2:**
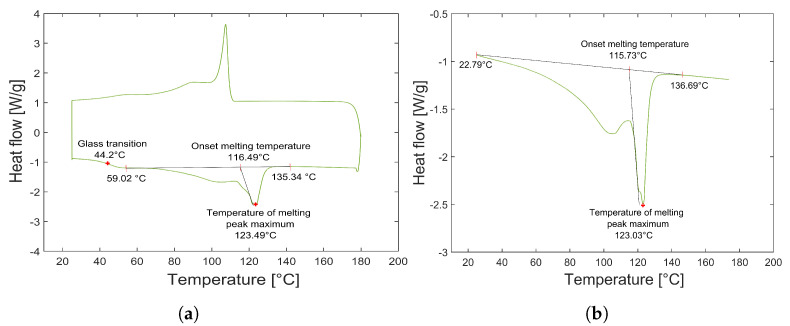
Differential scanning calorimetry (DSC) test plot for TPF1 (**a**) first heating ramp and (**b**) second heating ramp.

The DSC tests were carried out on all the thirteen selected films that met the required criteria. The $T_{m}$ of the TPFs obtained from the DSC test are summarized in Table 4. Among the TPFs supplied from Pontacol (TPF1 to TPF6), all TPFs except the TPF4 showed a typical melting behavior as that of a TP polymer. A sharp $T_{m}$ was observed for these TPFs during the DSC tests, therefore demonstrating a semi-crystalline nature. The DSC graph of the TPF1 is shown in Figure 2a,b.

The TPF7 supplied by L&L products did not show any clear $T_{m}$ during the DSC test, and thus, exhibiting an amorphous nature. The remaining TPFs supplied by AdhesiveInc (TPF8 to TPF13) did not show the desired behavior in the DSC tests. For some, the melting behavior was not clear, for others, either the melting ranges were way off as compared to the data provided by the supplier, or no $T_{m}$ was observed at all. For example, the TPF8 had two melting regions and no crystalline peaks were observed during the cooling phase; in case of the TPFs from TPF9, TPF10, TPF11, and TPF13, the $T_{m}$s were way off as compared to the data provided by the supplier and in the case of TPF12 no $T_{m}$ was found at all. The disagreement in the data provided by the supplier and the test results could be attributed to the different types of test standards used by the supplier and during this study.

#### 3.1.2. Reparability and Bonding

All the TPFs which did not have the desired melting behavior were not tested for reparability and bonding strength, except for TPF7 and TPF8. An exception was made for the TPF7 because of the high adhesion properties mentioned by the supplier were to be verified during the SLS tests. TPF8 was also tested because the two melting regions were inside the range of the set criteria. Therefore, the TPFs TPF4 and TPF9 to TPF13 were rejected and not used further in the study.

**Figure 3:**
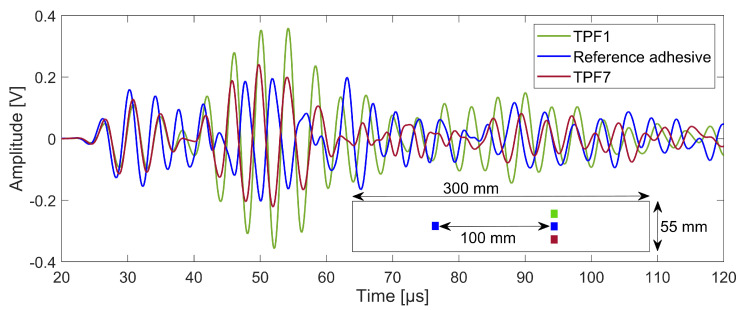
Lamb wave received by AUCTs bonded with the reference adhesive, TP1 and TP7.

Guided waves generated by an AUCT bonded by the reference adhesive and received on AUCTs bonded with the reference adhesive, TPF1 and TPF7 at 250 kHz in a pitch-catch configuration are shown in Figure 3. Only three cases have been demonstrated here representing each type of adhesive, i.e., the reference adhesive, the semi-crystalline TPF (TPF1), and the amorphous TPF (TPF7). It can be seen that the lowest time of flight corresponds to the AUCT bonded with the reference adhesive, while AUCTs bonded with TPF1 and TPF7 have almost equal time of flight. This is expected considering the distance between them and the actuator. The amplitude of the received signal is not same for the three cases, which might be because of the different stiffnesses and thicknesses of the adhesives used to bond the AUCTs. However, the aim was to demonstrate the ability of the AUCTs bonded with TPFs to receive guided waves to show that they are well bonded.

**Figure 4:**
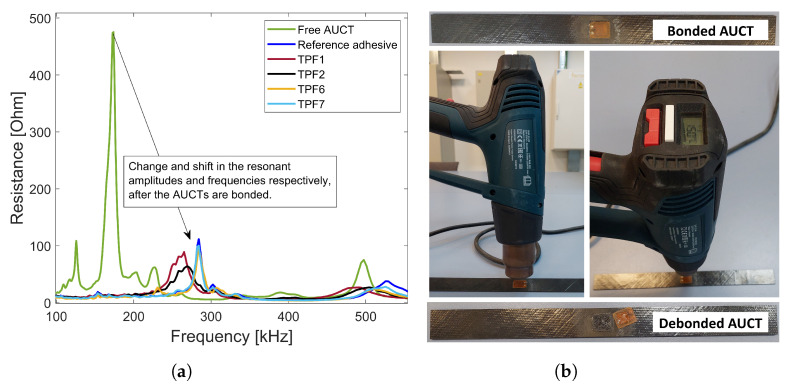
(**a**) EMI spectra comparison for free AUCTs and AUCTs bonded with different adhesives to check the bond quality (**b**) Bonded AUCT reparability setup by a temperature-controlled heat gun.

In Figure 4a, the EMI spectra of the reference adhesive, TPF1, TPF2, TPF6, and TPF7 are plotted along with the spectra of a free AUCT. The resonant frequency and the amplitude is different for AUCTs bonded with different adhesives because of the different stiffnesses and thicknesses of the adhesives used to bond the AUCTs. However, in each case, there is a large change in the resonant amplitude and frequency as compared to a free AUCT, which indicates that the AUCTs are well-bonded. The reparability of the TPFs was tested through a very simple setup. The adhesive bond line was heated by hot air using a temperature-controlled heat gun until the $T_{m}$ of the applied TPF. It was demonstrated that the AUCTs can be removed almost effortlessly without any damage to the host structure. The reparability setup is shown in Figure 4b.

**Figure 5:**
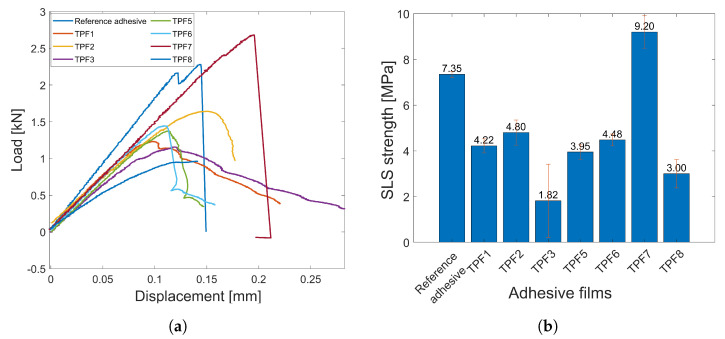
Single lap shear (SLS) test results. (**a**) Load vs. displacement curves for epoxy and different TPFs in the SLS test. (**b**) Mean and standard deviation of SLS strength of epoxy and different TPFs.

The SLS tests were carried out for the reference adhesive and all the TPFs which passed the reparability tests, i.e., TPF1–TPF3 and TPF5–TPF8. The objective was to evaluate and compare the bonding quality of the TPFs with that of the reference adhesive. The SLS test load-displacement curves for the reference adhesive and the TPFs are shown in Figure 5a, the SLS strength values are plotted in Figure 5b and summarized in Table 4.

The SLS strength of most of the TPFs is comparable to that of the reference adhesive except in case of TPF3 and TPF8. One of the adhesive films that stood out was TPF7; it was found that the SLS strength of this film was even higher than the reference adhesive, and hence, it can be concluded that it has really good adhesion properties as specified by the supplier and a good candidate to bond the AUCTs. The TPFs (TPF1, TPF2, TPF5, and TPF6) have acceptable SLS strength, whereas TPF3 and TPF8 have very low SLS strength. The reference adhesive which is an epoxy material and TPF7 both have a hard and brittle behavior, which is typically shown by thermoset polymers. This shows that TPF7 behaves more like a thermoset material but is reprocessable as well. The remaining TPFs shows a more ductile behavior as an elongation region can be observed before the break.

### 3.2. Sensor Defects

**Figure 6:**
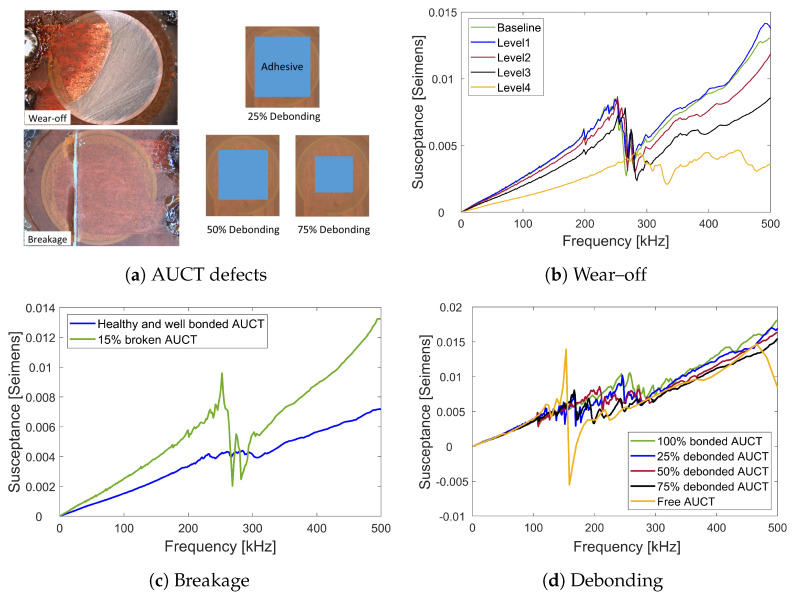
(**a**) Artificially created AUCT faults. Influence of AUCT faults on SS (**b**) wear–off (**c**) breakage and (**d**) debonding.

As mentioned, three commonly occurring AUCT defects were artificially created and their influence on SS was measured. The influence of these three defects, namely wear-off, AUCT breaking, and debonding, on the SS as compared to the baseline (healthy and well bonded) measurements, is shown in Figure 6b, Figure 6c and Figure 6d, respectively.

The AUCT wear-off causes a decrease in the slope of the SS. The percentage RMSD in slope between the healthy and three wear-off levels are 0.5%, 4%, and 38.6%, respectively. This means with severe AUCT degradation, there is a significant change in the SS slope. After each wear-off level, the amplitude of the first resonant peak decreases more, while the resonant frequency shifts towards the lower values. The deviation in the resonance amplitude and shift in the resonant frequency at severe wear-off is 34.1% and 7.2%, respectively. After severe wear-off, only the first resonant is clearly visible. This can be explained by the fact that the wear-off removes some of the piezoelectric material, and therefore, the admittance is the ability of AUCT to pass the current decreases significantly. The debonding produces a similar influence on the SS as that of the wear-off, i.e., the decrease in the SS slope, decrease in the first resonance amplitude, and shift of the resonant frequency towards the lower values as the amount of debonding increases. The percentage RMSD between the healthy and 25%, 50%, and 75% debonded AUCTs for slope, resonant amplitude, and resonant frequency are 2.9%, 12.5%, and 9.4%; 16.5%, 25.0%, and 30.4%; and 2.3%, 10.9%, and 14.9%, respectively. In case of the AUCT breakage, there is a significant change in the slope (30.5%) and the resonant peaks are not recognizable. This is explained by the fact that less piezoceramic material is now contributing to the voltage produced.

It can be seen that the defects can have a significant influence on the SS of the AUCTs. The SS curve characteristics that are affected significantly by the sensor defects are the slope, the magnitude of the first resonant peak, and the resonant frequency. Therefore, any significant change in these characteristics can indicate some damage or degradation on the AUCTs or the bonding layer.

### 3.3. Durability and Integrity

The bonded as well as free (not bonded) AUCTs were exposed to the AOEC tests, as described in Section 2.5. The AUCTs were bonded with the TPFs which have good bonding properties as obtained from the SLS test namely the reference adhesive, TPF1, TPF2, and TPF5–TPF7. The TPF3 and TPF8 were not tested in AOEC tests because of their low adhesion properties.

**Figure 7:**
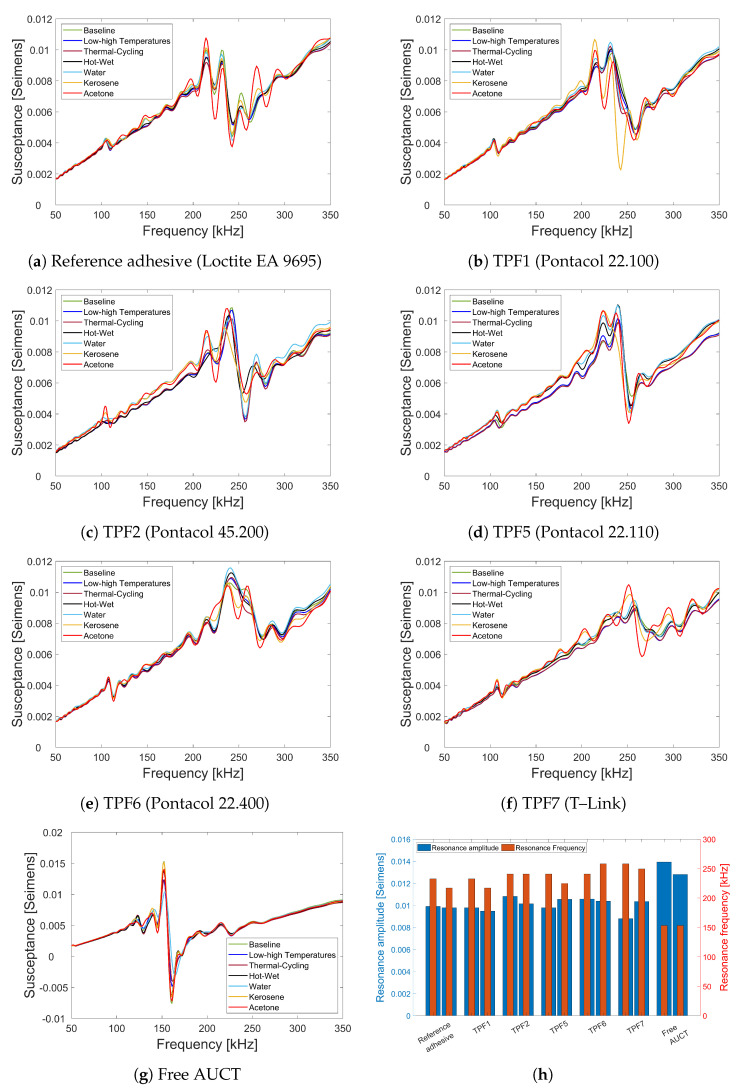
(**a**–**g**) SS for different adhesive bonded and free AUCTs after each AOEC test. (**h**) Absolute values of the first resonance amplitudes and frequencies before (left) and after (right) the AOEC tests.

**Figure 8:**
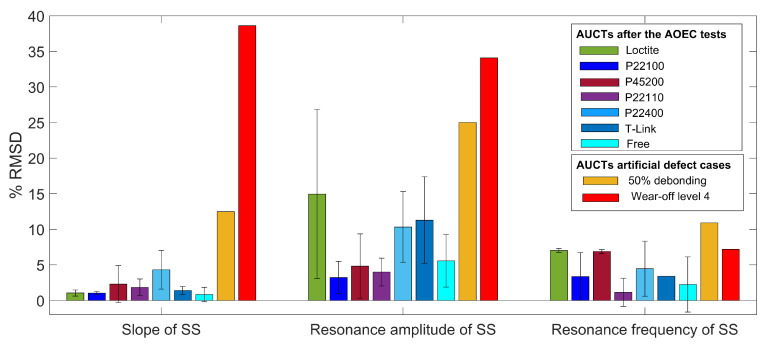
Comparison of the changes in the SS characteristics for artificially created defects vs. bonded and free AUCTs after the AOEC tests.

The SS plots of bonded and free AUCTs after each AOEC test are shown from Figure 7a–g. The absolute values of the first resonance amplitudes and their corresponding resonance frequencies before and after the AOEC tests for each adhesive and the free AUCT is shown in Figure 7h. In each case, the first bar represents the baseline values of the resonance amplitudes and frequencies and the second bar represents the values after the AOEC tests. In all of the cases a large deviation of the overall SS is not evident; however, some small changes can be observed. To quantify this change, the percent RMSDs of the SS slopes are calculated after each AOEC test and between the baseline and the final values as well. Figure 8 shows the mean percent RMSDs between the baseline and the final SS slopes for the three samples of each adhesive bonded and the free AUCTs. The change in the slope is small in each case, which indicates that the SS has not changed significantly. The least change in the SS slope has occurred in case of the free AUCTs with a mean change of less than 1%. In case of bonded AUCTs, the least change in the slope has occurred in the AUCTs bonded with TPF1 (Pontacol 22.100) with a mean change of about 1% and a very small standard deviation (std) of 0.2. The maximum change in the SS slope has occurred in the AUCTs bonded with the TPF5 (Pontacol 22.110) with a mean change of 4.3%; however, it is still not very high. For the rest of the AUCTs bonded with other TPFs, the mean change in the SS slope is very small, with the highest being 2.3% for TPF6 (Pontacol 22.400). If the changes in the slopes are compared between the AUCTs bonded with the reference adhesive and the TPFs, it can be seen that in case of TPF1 (Pontacol 22.100), the change in the SS slope is even smaller than that of the reference adhesive, while in case of other TPFs, the changes are similar to that of the reference adhesive except in case of TPF5 (Pontacol 22.110). Therefore, based on the SS slope changes, it can be said that TPF1 (Pontacol 22.100) outperforms the reference adhesive, while the remaining TPFs have a very similar performance to that of the reference adhesive.

The other two parameters that can give more insights about the specific changes that have occurred in the SS during the tests are the changes in the resonance characteristics, i.e., the amplitude and the frequency of the resonance. Figure 8 shows the mean percent RMSDs between the baseline and the final values of the first resonance amplitude and the frequency for the three samples of each adhesive bonded and the free AUCTs. In case of the bonded AUCTs, the least change in the mean resonance amplitude was observed in the AUCTs bonded with TPF1 (Pontacol 22.100), equal to 3.2%. A small change in the resonance amplitude can also observed in case of the AUCTs bonded with TPF2 (Pontacol 45.200) and TPF6 (Pontacol 22.400). However, in case of TPF5 (Pontacol 22.110) and TPF7 (T-Link), a significant change (greater than 10%) was observed in the mean resonant amplitude. The highest change in the mean resonance amplitude occurred in the AUCTs bonded with the reference adhesive equal to 14.9%. A small mean change of 5.6% in the resonance amplitude was observed in case of the free AUCTs. The change in the resonant frequency which is a more critical parameter is relatively small for all the bonded AUCTs, with the maximum being 7%. The least shift in the mean resonance frequency was observed in case of AUCTs bonded with the TPF2 (Pontacol 45.200), while the shift in the mean resonance frequency for TPF1 (Pontacol 22.100), TPF5 (Pontacol 22.110), and TPF7 (T-Link) is less than 4.5%. The highest shift in the mean resonance frequency was observed in the reference adhesive. In case of the free AUCTs, a small change in the resonance frequency was also observed, which might have been caused due to the degradation of the encapsulation material.

From Figure 8, it can be clearly seen that the changes caused in the SS characteristics, i.e., the slope, the resonant amplitude, and frequency due to the AUCT defects, are significantly large as compared to the changes that are caused due to the AOEC tests. Across all the SS characteristics, the highest changes are introduced by the AUCT defects, particularly in slope and resonant amplitude. Thus, it can be said that although there are changes caused in the AUCTs during the AOEC tests, they are significantly smaller than the defects caused due to AUCT defects, and therefore, no significant degradation has occurred in the AUCTs during the AOEC tests. If the changes that have occurred in the slope and resonance characteristics are considered together, then the best performing AUCTs are those which were bonded with the TPF1 (Pontacol 22.100). It outperforms all the other adhesive films in two of the performance parameters which is in agreement with the previous study carried by [8], while the worst performing adhesive is the reference adhesive, which has the lowest performance in terms of the change in the resonance characteristics. All the other TPFs have similar or better performance than that of the reference adhesive.

Another interesting thing that was observed during the AOEC tests was that most of the changes that occurred in the SS plots in all the bonded AUCTs occurred during the fluid susceptibility tests. This can be clearly seen in the SS plots for all the bonded AUCTs with different adhesive films. A noticeable change in the SS plots can be observed with the general trend of resonant frequency shifting to the left, as observed in case of artificially created defects and as also reported in [15,35]. This implies that all the adhesives used during this study do not exhibit an exceptional resistance to the studied fluids. However, all TPFs having semi-crystalline nature, i.e., TPF1, TPF2, TPF5, and TPF6 have a higher degree of fluid resistance, which generally speaking is due to their crystalline molecular structure that provides a barrier to fluid penetration. On the other hand, TPF7 is more susceptible to fluid absorption due to its amorphous structure, which allows fluids to penetrate the material more easily [37]. Although epoxy adhesives generally have good chemical resistance due to their cross-linked polymer chains, but in this study, it was not found to be excellent. However, in all the cases, the changes are small and do not represent any severe degradation of the adhesive layer or the AUCT. Furthermore, in case of the free AUCTs, some change can also be observed in the SS plots. Hence, the changes that have occurred in the SS of the bonded AUCTs are not necessarily due to the adhesive layer; they might also partially be due to the degradation of the encapsulation material (RTM 6) of the AUCTs themselves.

**Figure 9:**
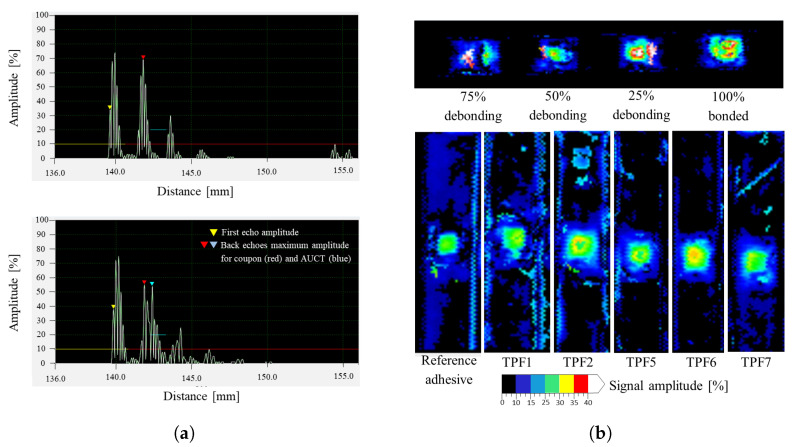
A-scans and C-scans of the artificially debonded and the AOEC-tested AUCTs. (**a**) A-scans of the locations were an AUCT is not bonded (**top**) and bonded (**bottom**). (**b**) C-scans of the AUCTs with artificially created debondings (**top**) and AOEC-tested AUCTs (**bottom**).

The results from the US inspections that were carried to further inspect the AOEC-tested AUCTs are shown in Figure 9a,b. Figure 9a shows A-scans of the locations on the coupons where the AUCT is not bonded (top) and where AUCT is bonded (bottom). A third back-wall echo in case of the A-scan where the AUCT is bonded can be observed; this back-wall echo is coming from the AUCT itself, showing that the adhesive layer is intact. If the AUCT was completely debonded, no such back-wall echo would be present as is the case in the A-scan, where no transducer is bonded. Figure 9b (top) shows the C-scans of the partially bonded AUCTs, where the colored scale represents the signal amplitude in %. The introduced percentage debonding from left to right is 75%, 50%, 25%, and 0%. It can be observed that there is some change in the C-scan amplitude because of the debondings. This change increases with the increase in the debonding percentage, although it is not necessarily proportional to the debondings that were introduced. Next, the C-scans of all the coupons tested in AOEC were taken to find if any significant debondings can be found. The C-scans of the AOEC-tested coupons are shown in Figure 9b (bottom). It can be seen that unlike the debonding cases, there is not a significant change in the C-scan signal amplitude for the AOEC-tested AUCTs. Therefore, it seems to indicate that significant debondings or degradation have not occurred on the AUCTs bonded with different adhesives during the AOEC tests. However, it is important to mention that the US equipment used is limited by its resolution to find very small debondings or adhesive degradation if it exists. However, in the scope of this study, the focus is more on the finding any significant changes that can occur during the AOEC tests, for which this resolution is sufficient.

## 4. Conclusions and Outlook

A new efficient method of integrating PCTs to TP composite structures was proposed by using TPFs rather than epoxy adhesives. The TPFs were chosen based on certain criteria and characterized with standard tests. The bonding ability of the chosen TPFs was checked by EMI and lamb-wave measurements. The reparability, which is one of the main advantages of TP adhesives, was demonstrated through a very simple setup. To demonstrate the durability of the proposed bonding method in aeronautical applications, the AUCTs were bonded to TP coupons through the chosen TPFs and put through standard AOEC tests. It was shown that the changes produced in the AUCT characteristics during the AOEC tests were significantly smaller than the changes caused due to AUCT defects. Thus, no significant degradation occurred in the AUCT during the AOEC tests. The most critical AOEC tests for all the adhesive films whether the epoxy reference film or the chosen TPFs were the fluid susceptibility tests of Kerosene and Acetone, which introduced the maximum changes in the SS characteristics. However, such scenarios of AUCTs getting immersed in fluids such as Kerosene and Acetone are less likely to occur in real-life applications. Nevertheless, any changes that can occur in the AUCT characteristics because of these conditions should be taken into account in the SHM system. Taking these changes into account can also help the SHM designer to set a threshold to distinguish between the changes in the guided wave signal because of the sensor and structure in order to avoid any false alarms.

After comparing the performance of the bonded AUCTs in the AOEC tests, it was seen that some TPFs outperformed the reference epoxy film, while other TPFs had a similar performance, like that of the reference epoxy film. Thus, the proposed method of bonding the AUCTs is more efficient, easy, and reliable than the current state-of-the-art method of bonding AUCTs with epoxy glue.

However, this study is limited to a particular type of epoxy film adhesive and special state-of-the-art PCT transducers. Although they represent the state-of-the-art, it may be possible that other types of epoxy adhesives and PCTs may have better or worse performance in the AOEC tests performed in this study. This study could be extended to other types of adhesives to establish if the TPFs have better performance with respect to other adhesives or not. In addition to EMI methodology employed in this study to measure the changes in the AUCT characteristics, other methods, such as changes in the guided wave signals and out-of-plane velocity, could be performed, which can give more insights into the changes that can occur in the adhesive layer and the AUCT characteristics during the AOEC tests.

In the current study, the AUCT have been bonded to the composite specimen by oven heating and vacuum bagging. This process can be time-consuming and may require heating the entire structure, which can cause thermal degradation to the structure. To overcome these drawbacks, an easier, faster, and economic method of bonding AUCTs to TP composite structure using local induction heating is currently being investigated by the authors. Furthermore, it is very important to study the mechanical performance of the selected TPFs as compared to the epoxy adhesives used to bond the AUCTs. Therefore, the mechanical performance of the AUCTs bonded with the TPFs with oven and induction heating will be studied in great detail through different mechanical tests, such as tensile-tensile fatigue and static and cyclic flexural tests, and compared with that of the epoxy adhesives.

## Figures and Tables

**Table 1:** Comparison of TP and thermoset based adhesives.

Propery/Parameter	Epoxy (Thermoset Adhesives)	TP Adhesives
Storage	Refrigeration maybe required	No refrigeration required, can be stored at room temperature
Shelf life at room temperature	Short	Long
Cycle times	Longer	Shorter
Reparability	Difficult	Yes
Weldability	No	Yes

**Table 2:** TPFs selected based on the defined criteria.

Adhesive Film	Material	Supplier
22.100 (TPF1)	Polyolefin (POF)	Pontacol
45.200 (TPF2)	POF/Copolyamide (Multi-layer)	
45.350 (TPF3)	POF/ Polyurethane (PUT) (Multi-layer)	
46.302 (TPF4)	PUT	
22.110 (TPF5)	Low-density polyethylene	
22.400 (TPF6)	High-density polyethylene	
T-Link (TPF7)	Unknown	L&L Products
PAF-130 (TPF8)	Polyester	AdhesiveInc.
NAF-605 (TPF9)	Polyamide	
UAF-438 (TPF10)	PUT	
UAF-472 (TPF11)		
EXF-951 (TPF12)		
UAF-410 (TPF13)		

**Table 3:** Standard AOEC tests carried out on the AUCTs.

Integrity Test	Standard	Parameters	Equipment
Temperature	RTCA DO-160E-4.5.2 RTCA DO-160E-4.5.4	Operating high temperature at 70 °C for 2 h Operating low temperature at −55 °C for 2 h	
Thermal cycling	RTCA DO-160E-5.3.1	Shock from −55 °C to 70 °C in 25 min.	Dycometal CCK-70/80 Climate Chamber
Hot-Wet	RTCA DO-160E-6.3.1	50 °C to 38 °C (5 °C/min) RH 95% to 85% for 48 h	
Fluid susceptibility	RTCA DO-160E-11.4.2	Immersed in water for 168 h at 70 °C, Kerosene for 24 h at 40 °C and Acetone for 24 h at Room temperature 24 °C.	

**Table 4:** DSC and SLS test results for different TPFs.

Adhesive Film	Average $T_{m}$ [°C]	Reparability	Average SLS [MPa]
Reference adhesive	-	No	7.35
TPF1	123.0	Yes	4.22
TPF2	122.4	Yes	4.80
TPF3	122.4	Yes	1.82
TPF4	-	No	Not tested
TPF5	116.2	Yes	3.95
TPF6	127.9	Yes	4.48
TPF7	-	Yes	9.20
TPF8	121.9	Yes	3.00
TPF9	75.5	Not tested	Not tested
TPF10	49.2		
TPF11	48.4		
TPF12	-		
TPF13	47.0		

## Data Availability

Data are available on request.
